# *Ex vivo* study of neuroinvasive and neurotropic viruses: what is current and what is next

**DOI:** 10.1093/femsre/fuaf024

**Published:** 2025-06-11

**Authors:** Alexandre Lalande, Cyrille Mathieu

**Affiliations:** CIRI, Centre International de Recherche en Infectiologie, NeuroInvasion TROpism and VIRal Encephalitis Team, Univ Lyon, Inserm, U1111, Université Claude Bernard Lyon 1, CNRS, UMR5308, ENS de Lyon, 21 Avenue Tony Garnier, F-69007 Lyon, France; CIRI, Centre International de Recherche en Infectiologie, NeuroInvasion TROpism and VIRal Encephalitis Team, Univ Lyon, Inserm, U1111, Université Claude Bernard Lyon 1, CNRS, UMR5308, ENS de Lyon, 21 Avenue Tony Garnier, F-69007 Lyon, France

**Keywords:** neuroinvasion, neurotropism, virus, *ex vivo*, organoid, organotypic culture

## Abstract

Numerous pathogens, including viruses, enter the central nervous system and cause neurological disorders, such as encephalitis. Viruses are the main etiologic agents of such neurological diseases, and some of them cause a high death toll worldwide. Our knowledge about neuroinvasive and encephalitogenic virus infections is still limited due to the relative inaccessibility of the brain. To mitigate this shortcoming, neural *ex vivo* models have been developed and turned out to be of paramount importance for understanding neuroinvasive and neurotropic viruses. In this review, we describe the major *ex vivo* models for the central nervous system, including neural cultures, brain organoids, and organotypic brain cultures. We highlight the key findings from these models and illustrate how these models inform on viral processes, including neurotropism, neuroinvasion, and neurovirulence. We discuss the limitations of *ex vivo* models, highlight ongoing progress, and outline next-generation *ex vivo* models for virus research at the interface of neuroscience and infectious diseases.

## Teaser

Knowledge about viruses infecting the brain has been primarily acquired with neural cultures, but more relevant 3D *ex vivo* models such as brain organoids and organotypic brain slice cultures are increasingly used to decipher neuroinvasion, neurotropism, and neurovirulence of these pathogens.

## Introduction

For viral pathogens, neuroinvasiveness is usually defined as the capacity to enter the nervous system, while neurotropism is related to the ability to infect and replicate in neural cells. Neurovirulence is a notion linked to central nervous system (CNS) disease manifestation caused by a virus, independently of neuroinvasion or neurotropism (Bauer et al. 2022). Encephalitic viruses are thus neurovirulent because they cause CNS pathology, but all neuroinvasive viruses are not necessarily neurotropic or neurovirulent. Likewise, a virus can be neurotropic in cell culture experiments, but can be unable to enter the nervous system (i.e. be non-neuroinvasive). Neuroinvasive and encephalitic viruses gather a myriad of pathogens from different families, such as herpes simplex virus type 1 and cytomegaloviruses (HSV-1 and CMV, family *Orthoherpesviridae*), Borna disease virus 1 (BoDV-1, family *Bornaviridae*), rabies virus (RABV, family *Rhabdoviridae*), enterovirus A71 and D68 (EV-A71 and -D68, family *Picornaviridae*), West Nile, Dengue, Zika, and tick-borne encephalitis virus (WNV, DENV, ZIKV, and TBEV, family *Flaviviridae*), La Crosse virus (LACV, family *Peribunyaviridae*), human immunodeficiency virus (HIV, family *Retroviridae*), canine distemper, measles, Hendra and Nipah virus (CDV, MeV, HeV, and NiV, family *Paramyxoviridae*), to cite a few. Encephalitis, which is classically defined as the inflammation of the brain, can be the result of a direct brain infection or indirect affection following infection outside the CNS. Numerous viruses are thought to commonly reach the CNS, accidentally or as a result of a genuine neurotropism, transiently/acutely or chronically/persistently, actively replicating or not (Ludlow et al. 2016, Bookstaver et al. 2017). However, what is known about these viruses mainly comes from data obtained in animals or after symptoms onset and post-mortem. Thus, a knowledge gap exists regarding the early, asymptomatic, and prodromal stages of viral encephalitis, but also regarding neuropathology progression at the organ, cellular, and molecular levels, because of the relatively inaccessible nature of the CNS. To investigate these aspects, relevant models are needed and researchers have used three main types of *ex vivo* systems of increasing complexity, namely neural (poly)cultures, organoids, and organotypic cultures. Their primary goal is to mimic the tissue/organ microenvironment, and regarding neurovirulent viruses, to ultimately model and decipher notably viral possible entry routes into the CNS, neurotropism, dissemination, virus–host cell interactions, and viral evolution.

Viruses are the main cause of encephalitis, which often co-occurs with viral meningitis. Although the cause of many encephalitic cases remains unknown, viruses are the etiologic agent of 70% of confirmed cases. Viral encephalitis still causes high morbidity (3.5 to 7.5 per 100 000 people) and mortality (up to 90%–100% depending on the pathogen and outbreaks) worldwide (Venkatesan 2015, Ludlow et al. 2016, Bohmwald et al. 2021, Said and Kang 2024). Although specificities in pathophysiological features exist depending on the virus, encephalitis is essentially an inflammation of the brain parenchyma that causes brain swelling and neurological damage, sometimes associated with vasculitis. Broadly speaking, cerebral edema, vascular congestion, thrombosis, hemorrhage, necrosis, immune cell infiltration and associated inflammatory response can regularly be observed. Brain regions and neural cells can be differentially infected depending on the virus (Dahm et al. 2016, Bohmwald et al. 2021). Encephalitic viruses can reach the CNS by different routes. They can use anterograde or retrograde axonal transports from peripheral neurons (e.g. motor or olfactory neurons, for RABV for example) (Lycke and Tsiang 1987). Access can also occur hematogenously by the crossing of the blood–brain/cerebrospinal fluid or meningeal–cerebrospinal fluid barrier, via direct infection of endothelial cells, compromising the barrier, or via infiltration of virus-carrying immune cells, in the case of WNV for instance (Wang et al. 2004, Verma et al. 2010). For a certain number of pathogens such as MeV, NiV, or severe acute respiratory syndrome coronavirus 2 (SARS-CoV-2), entry routes into the CNS during natural infection are still not completely deciphered (Griffin et al. 2012, Koyuncu et al. 2013, Bauer et al. 2022, Jagst et al. 2024).

**Figure 1.**
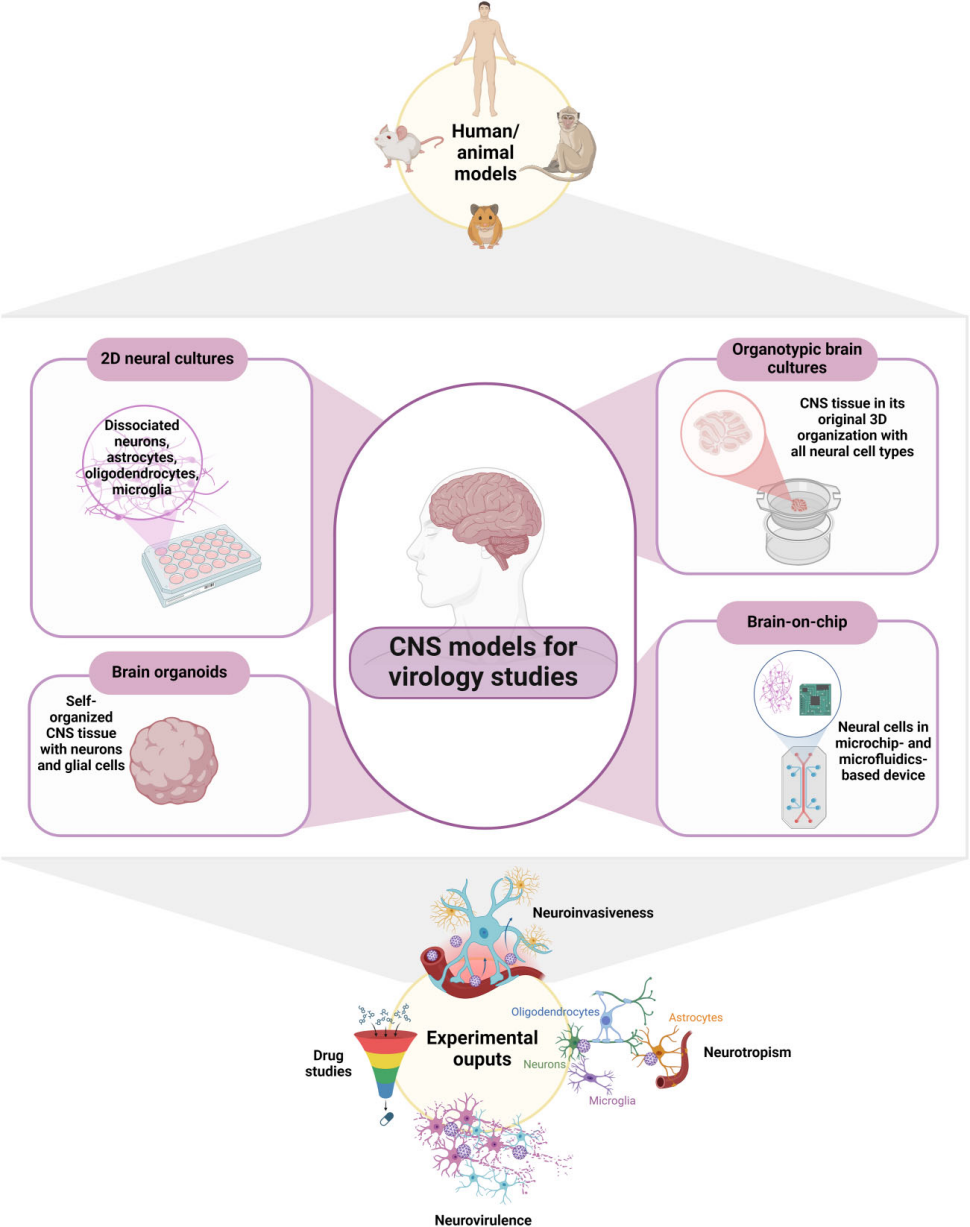
Overview of the different CNS models used to address questions related to neuroinvasive and neurotropic viruses. Created in BioRender.com.

As stated above, three important notions regarding these viruses infecting the CNS are neuroinvasiveness, neurotropism, and neurovirulence, which are often used interchangeably in the literature although they refer to distinct meanings, a fact particularly highlighted during the SARS-CoV-2 pandemic (for a review, see Bauer et al. 2022). Actually, these are central questions that are modeled in *in vitro* and *ex vivo* models for the study of encephalitic viruses: How does a virus enter the nervous system (neuroinvasiveness), which cells of the nervous system are infected by this virus (neurotropism), and how does the virus cause nervous system pathology (neurovirulence) (Fig. 1)? We detail here how CNS models are used to study viral neuroinfections by highlighting their properties and key findings about different pathogens, and what will be the future developments for these systems.

## Two-dimensional models: neural cultures

Classical neural (poly)cultures consist of two-dimensional (2D) cultures of individual or mixed neural cell types (neurons, astrocytes, oligodendrocytes, and microglia). They can be obtained from different parts of the CNS (spinal cord, brain substructures such as cortex, cerebellum, hippocampus, brainstem, etc.). Various methods to generate these cultures and subsequently use them in infection studies have been developed over the years. The most straightforward method for generating these cultures is the isolation and culture of a single cell type from embryonic, fetal, or neonatal animal brains (Banker and Cowan 1977, Brewer et al. 1993, Ahlemeyer and Baumgart-Vogt 2005, Brewer and Torricelli 2007). Alternatively, neural stem/progenitor cells can be isolated and cultured as adherent monolayer cultures or neurospheres (i.e. free-floating neuroprogenitor cells clusters), which can self-renew, proliferate, and be passaged in long-term culture. They remain in an undifferentiated or early differentiated state and can thus then be differentiated into neurons, astrocytes, and oligodendrocytes (Liem et al. 1995, Gobbel et al. 2003, Yan et al. 2005, Brewer and Torricelli 2007, Barak et al. 2022). Such cultures can also be obtained from embryonic stem cells (ESC) (Yan et al. 2005) or induced pluripotent stem cells (iPSCs) (Barak et al. 2022).

**Figure 2.**
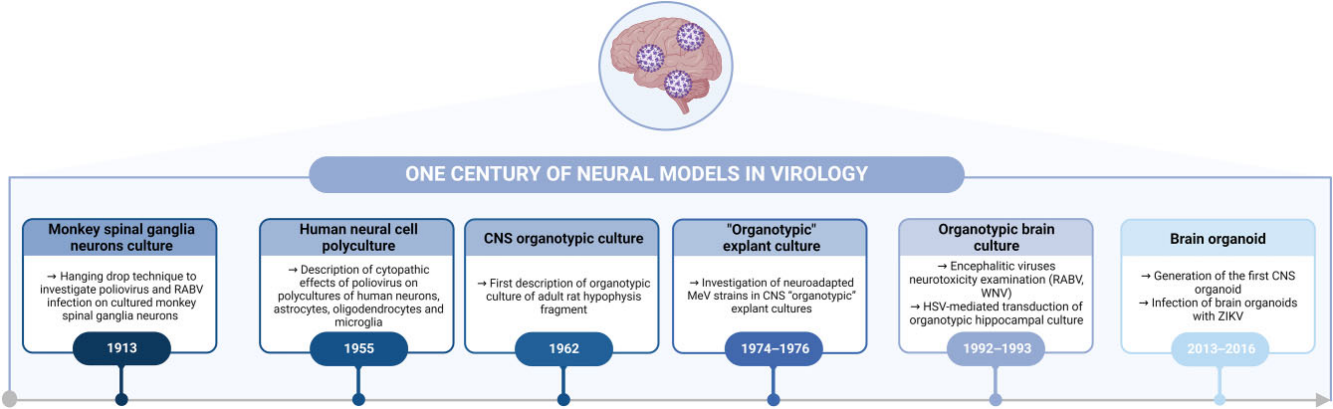
Timeline summarizing milestones in the implementation of neural *ex vivo* models in virology research. Created in BioRender.com.

These cultures have been used for decades to study encephalitogenic and other neurovirulent viruses. Almost 70 years ago, cytopathological effects of poliomyelitis virus upon infection of isolated human neural cells were described (Hogue et al. 1955), and in the late 1960s, cultured human and murine glial cells were reported to sustain long-term production of highly encephalitogenic arboviruses without cytopathic effect (Illavia and Webb 1969) (Fig. 2). Neural cultures are particularly useful tools because they are a simple reductionist system that is relatively easy to implement and analyze, being rather well defined in terms of culture conditions (medium, oxygen needs, etc.). They enable working without the influence of hormonal, vascular, and immune/inflammatory factors facilitating intracellular and cell-to-cell observation (Brewer and Torricelli 2007). To go further, they allow the study of various aspects of encephalitic neuroinvasive viruses, from neural cell permissiveness, intracellular transport of nucleocapsids, cytopathic effects, viral persistence, to viral spread between neural cells.

### *Bornaviridae*: BoDV-1

BoDV-1 is a prototypical example of a highly neurotropic virus, which causes severe encephalitis, mainly in horses and sheep, but also in humans, although little is known regarding pathogenesis in the latter (Jungbäck et al. 2025). BoDV-1 was shown to infect and replicate in human neural stem/progenitor cells without altering their survival. However, in mixed neurons-astrocytes cultures, it highly impaired the survival of neurons newly generated upon differentiation of these progenitor cells, by inducing Caspase-3-mediated apoptosis (Brnic et al. 2012). Surprisingly, infection of the main target of the virus, hippocampal neurons, does not impair neuronal survival (Hans et al. 2004). Infection of neurons grown on multielectrode arrays revealed no impact on spontaneous neuronal activity. It showed, however, an impairment of synaptic plasticity, because pharmacological induction of increased synaptic efficacy by bicuculline causes a rise in neuronal burst frequency (reflective of synaptic activity), but this high level of network activity did not last after drug removal for BoDV-1-infected neurons that returned to basal levels, contrary to non-infected ones whose increased activity lasted for several hours (Volmer et al. 2007, Prat et al. 2009). Another impact of BoDV-1 on neuron physiology, as shown following infection of primary rat neurons, is the increase of the proportion of DNA double-strand breaks, which serve as a docking platform for the virus replication organelle, resulting in impaired neuronal firing (Marty et al. 2021). The literature provides discordant results concerning BoDV-1 cytopathicity and persistence in neural cells (Ovanesov et al. 2008a,b, Brnic et al. 2012, Jungbäck et al. 2025). As a matter of fact, BoDV-1 pathogenesis particularly highlights the discrepancies that can exist between *in vivo* and *ex vivo* infection, but also the utility of the neural cultures to investigate contradictory observations. BoDV-1 is non-cytopathic and non-cytolytic in cultured rat neurons; however, in infected newborn animals, neuronal death is observed (Weissenböck et al. 2000, Hans et al. 2004). In addition, infection of adult rats is characterized by strong neurotropism associated with neuronal destruction and leads to severe meningoencephalitis, as observed for human infection (Jungbäck et al. 2025). Neural cultures showed that neuronal death actually occurs during differentiation as a result of viral interference on pathways important for neuronal maturation (Brnic et al. 2012). On the other hand, BoDV-1 can also persist in the brain of animals without impairing neurons (Lipkin et al. 2011, Nobach et al. 2015). Microglia activation has also been proposed as a mechanism responsible for neuronal death in neonatal infection (Ovanesov et al. 2008a,b).

More specifically, sole expression of BoDV-1 phosphoprotein in human neural progenitor cultures inhibited neurogenesis, a phenotype correlated with decreased expression of genes involved in neuronal differentiation (Scordel et al. 2015). In-depth study of non-cytolytic BoDV-1 persistence revealed that it depends on the expression of the viral non-structural protein X, which localizes in the nucleus and in mitochondria and inhibits the induction of apoptosis in neurons (Poenisch et al. 2009). This led to investigating the promising therapeutic potential of BoDV-1 X as a neuroprotective protein in the context of neurodegenerative pathologies. Indeed, using microfluidic cultures of primary neurons, the protein was shown to protect neurons against neurogenerative insults, notably by preventing axonal fragmentation and enhancing mitochondrial filamentation. In rotenone-treated neurons, X conferred protection against oxidative stress (Szelechowski et al. 2014). Engineering a protein with improved mitochondrial localization led to increased neuroprotection (Ferré et al. 2016). Further, expression in primary motor neurons from a murine model of amyotrophic lateral sclerosis, characterized by mitochondrial dysfunction leading notably to decreased ATP production,of the domain of X responsible for its neuroprotective effects, restored ATP levels. Likewise, *in vivo* treatment of this murine model with X or domain thereof delayed symptoms onset and improved disease progression, with notably improved motor performance and motor neuron survival (Tournezy et al. 2024).

### *Orthoherpesviridae*: CMV, HTLV-1, and HSV-1

By contrast, human CMV (HCMV) impairs neuronal differentiation and is cytopathic to precursor cells, as demonstrated with neurosphere cultures from human fetus brains, but also to differentiated neurons and astrocytes (van Den Pol et al. 1999, Odeberg et al. 2006). Similarly, HIV infection of human neurospheres revealed that their differentiation ability was not impaired but newly generated neurons showed signs of injury and decreased expression of neuronal markers (McCarthy et al. 2006). In the same family, human T-lymphotropic virus 1 (HTLV-1) can infect the CNS and cause neuroinflammation and highly rare cases of encephalitis, although the consequences of HTLV-1 neuroinfection may be underestimated (Costa et al. 2012, Rocamonde et al. 2023a,b). Little is known about the neurotropism of HTLV-1, and it was recently shown to primarily target neurons in human iPSCs-derived neural polycultures and in the brain of primates naturally infected (Rocamonde et al. 2023a). CNS infection by HSV-1, the most common cause of encephalitis, was also modeled using human iPSCs-derived neurons. This system was used for demonstrating their susceptibility to infection by this virus that can enter a quiescent state in these cultures but not in progenitor cells, for comparing the changes in gene expression, cellular functions, and epigenetic marks between acute, quiescent/lytic, and latent infection of neurons, and for comparing antiviral drugs (D'Aiuto et al. 2015, 2019).

### *Rhabdoviridae*: RABV

On the contrary, RABV is a well-known zoonotic agent causing a well-documented encephalitis, and *ex vivo* studies using stem cell-derived human forebrain-type neuron/astrocyte cultures, combined with microfluidics, demonstrated induction of inflammatory cytokines, absence of neuronal death, and axonal trafficking of the virus in the neuronal network upon infection (Sundaramoorthy et al. 2020a). Infection of murine primary neurons revealed a selective viral-induced axonal and dendritic degeneration that hinders virus spread between connected neurons (Sundaramoorthy et al. 2020b).

### *Paramyxoviridae*: MeV and NiV

Primary neural cultures have been widely used to study important aspects of MeV, a paramyxovirus causing rare but serious acute or delayed encephalitis, with respect to its neuroinvasive properties, especially interneuronal and intraneuronal dissemination (Young and Rall 2009). Using primary cultures of embryonic hippocampal neurons, MeV (vaccine strain) cell-to-cell spread was shown to be receptor-independent and to require direct cell-cell contact but not syncytia formation, as reported in the brain of patients with MeV encephalitis. In patient brains, large syncytia are not commonly observed compared with what is seen for non-neuronal cells, leading to the hypothesis of a trans-synaptic transmission (Lawrence et al. 2000). Infection of similar hippocampal neuron cultures revealed that the well-known antiviral protein Tetherin surprisingly promotes MeV neuronal infection (Miller et al. 2021). In pure cultures of neurons and cocultures of murine neurons and astrocytes, it was shown that MeV seemed unable to infect astrocytes directly, but that neuron-astrocyte contact was needed. In astrocytes, MeV can replicate and disseminate without formation of syncytia and production of infectious units, which may be related to their natural ability to self-organize in syncytial-like networks. Moreover, neuron-neuron and astrocyte-astrocyte spread was independent of a high affinity receptor and required cell membrane fusion, contrary to neuron-astrocyte spread that may rely on the release of infectious units in the synaptic cleft (Poelaert et al. 2021). Note, however, that MeV remains a primate-restricted virus, because outbreaks in both human and non-human primates are reported (MacArthur et al. 1979, Choi et al. 1999, Dogadov et al. 2023), which may lead to some variation in the infection behavior between species used to model infection.

These models, along with three-dimensional (3D) models to some extent, as described below, have been used and optimized for studying neurotropic/neuroinvasive viruses such as BoDV-1 or MeV, and further validated for emergent pathogens such as NiV or SARS-CoV-2. For NiV, infection of primary human olfactory neurons revealed efficient viral replication and a cytopathic effect (Borisevich et al. 2017, Ozdener et al. 2023), arguing for the existence of an olfactory route for Henipavirus neuroinvasion, as already suggested in a hamster *in vivo* model (Munster et al. 2012). This model recapitulates some key features of the human olfactory epithelium, such as heterogeneity in olfactory cell population, expression of olfactory receptors, and responses to odorant cues (Borisevich et al. 2017, Ozdener et al. 2023).

### *Flaviviridae*: TBEV and ZIKV

In the *Flaviviridae* family, TBEV is well described to cause neurological pathology but little is known about the molecular interplays governing viral tropism and susceptibility of neural cells. In this regard, infection of human neural progenitor-derived neuronal-glial cultures phenocopies natural human brain infection with predominant tropism for neurons inducing neuronal death, and astrogliosis. The authors demonstrated that distinct susceptibility in neurons and astrocytes was linked to differential antiviral capacities, with astrocytes being able to upregulate more immune genes and more strongly than neurons (Fares et al. 2020). With respect to TBEV-induced neuronal death, transcriptomic analysis in the same model revealed an upregulation of pyroptosis- and apoptosis-related genes upon infection (Fares et al. 2021). Similarly to BoDV-1, study of human primary neural progenitor cells infected with ZIKV indicated that neurogenesis and differentiation were stimulated upon infection, via notably the aberrant activation of the Notch pathway, inducing premature differentiation. Progenitor cells were more permissive to the infection than cells differentiated into neurons and astrocytes, phenotypes driven by the mounting of a robust innate immune antiviral response in more differentiated cells. Connecting these data to natural pregnancy infection, they may explain the drastic negative impacts on fetal brain development (leading notably to microcephaly and other neurological disorders), via the targeting of neural progenitors and dysregulation of neurogenesis (Ferraris et al. 2019).

### *Coronaviridae*: SARS-CoV-2

Regarding SARS-CoV-2, there were conflicting data on whether it commonly targets the brain before the Omicron variant, especially because most of them come from post-mortem analysis that are a picture of the infection endpoint and resulting damages, which do not necessarily reflect the previous stages of the infection (Matschke et al. 2020, Ferren et al. 2021, Meinhardt et al. 2021, Solomon 2021, Song et al. 2021, Bauer et al. 2022, Beckman et al. 2022, Stein et al. 2022). Be that as it may, SARS-CoV-2-related CNS complications and (rare) cases of encephalitis exist (Vandervorst et al. 2020, Valencia Sanchez et al. 2021). Investigation of SARS-CoV-2 neurovirulence in primary murine neurons and human ESC-derived cortical neuron cultures revealed neuronal fusion induced by infection that resulted in impaired synaptic activity (Martínez-Mármol et al. 2023).

### Drawbacks of the model

Despite the simplicity to obtain and to use them to investigate important aspects of virus biology, the representativeness of neural cultures regarding CNS physiology is debatable (Table 1). As a cell culture technique, neural cells are kept out of their tissue and organ context, meaning that initial 3D configuration and cellular interactions are lost and recreated in two dimensions. Moreover, for cultures of a single cell type, neurons in particular, purity can be challenging because glial cells can proliferate at a high rate and overgrow the culture (Altman 1963, Lesslich et al. 2022). In polycultures, having a representative ratio of the four main CNS cell types is also not trivial (Lesslich et al. 2022). For example, depending on the protocol used, embryonic cultures can typically yield 60%–95% neurons and 5%–40% astrocytes, or 40% neurons, 50% astrocytes, and 10% microglia, while adult cultures consist of 80% neurons, 10% oligodendrocytes, 5% microglia, and 5% astrocytes (Gao et al. 2002, Patel and Brewer 2003, Brewer and Torricelli 2007, Gao 2012, Goshi et al. 2020). Cell maturity (and resulting ability in terms of neuronal electrical activity or glial phagocytosis for example), differentiation, neuron myelination, formation and abundance of synapses, etc., can also vary greatly between the vast number of available protocols. Animal cultures usually require waiting ~2 weeks before using them for infection studies, and astrocytes seem to show an activation profile that may impede infection success and bias observations (Thomson et al. 2008, Kleinsimlinghaus et al. 2013, Gilmour et al. 2019). iPSCs have proven their potential for the generation of relevant neural cultures, especially for overcoming the difficulty to have access to primary human neural cells and for obtaining cultures of high quality and purity, but are still an expensive and time-consuming method. They can virtually generate all cell types of the CNS and thus have been used to produce pure and defined mixed neural cultures (Guttikonda et al. 2021, Sato et al. 2021, Barak et al. 2022). Neural cultures also usually lack blood–brain barrier (BBB, encompassing endothelial cells and pericytes) and vasculature more broadly speaking, choroid plexuses, and immune system, which are important elements in the neuropathogenesis of neurovirulent and encephalitogenic viruses.

**Table 1.** Advantages and limitations of the main neural models used to study neuroinvasive and neurotropic viruses.

	Neural cultures	Brain organoids	Organotypic brain cultures
Advantages	Easy; quick; inexpensive; long-term culture; rather well characterized	3R compliance; 3D cultures; patient-specific; starting material easy to obtain even from humans; better reflects *in vivo* cell identity; model of any brain region; more predictive than cell cultures	3R compliance; 3D cultures in its original tissue configuration; all cell types; easy to obtain; easy to infect and analyze; more predictive than cell cultures
Limitations	Cells out of their physiological context; difference between young and old animal; access to human cells difficult; potential lack of cell types; lack of vascularization, immune system, and blood–brain barrier; low representativeness of physiological neural populations; heterogeneity in protocols	Expensive; long and not easy to obtain; high variability; usually lack microglia, vascularization, immune system, and blood–brain barrier; infection and analysis somewhat tricky; necrosis in the center; heterogeneity in protocols; imperfect replication of the precise brain cellular composition, diversity, and structural organization brain	Difference between young and old animal; access to human organ/sample difficult; lack of vascularization, immune system, and blood–brain barrier; slicing-induced astrogliosis; possible proliferation of glial cells; hard to standardize human slices

## 3D microphysiological models: towards complex brain organoids

Another application of iPSCs is the generation of brain organoids, which are self-assembled microphysiological 3D culture systems composed of neuronal and glial cells recapitulating aspects of the brain (Lancaster et al. 2013, Lancaster and Knoblich 2014, Barreras et al. 2023, Birtele et al. 2025). They can be obtained from human and animal cells, which is advantageous for studying zoonotic neurotropic viruses from different host species (Pain et al. 2021, Kim et al. 2025). Unguided protocols spontaneously produce organoids, giving rise to heterogeneous structures. Alternatively, guided protocols use specific patterning and growth factors to generate more organized organoids that are representative of a given brain region (e.g. cortex, hippocampus, or midbrain) (Fan et al. 2022, Barreras et al. 2023). Their use for studying encephalitogenic viruses is largely less widespread than that of neural cultures, although they allow to address questions related to neural permissiveness, viral neurotropism, viral spread, cytopathic effects, impacts on organoid gene expression, organization, size and innate immune response upon infection, and drug testing (Depla et al. 2022, Barreras et al. 2023). Their major interest is the possibility to genetically modify iPSCs to study gene mutations and their impact on viral infection in a complex cellular model. In addition, they can be derived from somatic cells easily accessible from patients (Beghini et al. 2024).

### *Flaviviridae*: ZIKV and DENV

Cerebral organoids have been notably used to study ZIKV-induced microcephaly, and showed reduced size of organoids, disorganization with less ventricles and loss of progenitor cells upon infection (Garcez et al. 2016, Qian et al. 2016, Antonucci and Gehrke 2019). In the study of Garcez et al., organoid growth impairment was specific to ZIKV because infection with DENV2 (16681 strain), another closely related Flavivirus, did not impact organoid size (Garcez et al. 2016). In particular, ZIKV and DENV show major pathological differences in human brain organoids, with ZIKV infection inducing apoptosis and defects of growth and folding in cortical organoids, while DENV infection does not (Li et al. 2017b). ZIKV preferentially infects neural progenitor cells, consistent with developmental defects reported when infections occur during pregnancy (Garcez et al. 2016, Tang et al. 2016). The AXL protein has been proposed to be a receptor for ZIKV in the brain. However, genetic ablation of *AXL* by TALENs and CRISPR genome editing technology in iPSCs, then differentiated into neural progenitor cells or brain organoids, did not prevent infection and death of the cells (Wells et al. 2016). This work was further supported by cell culture studies showing that AXL mediates ZIKV entry into human astrocytes and microglia but not neural progenitors, and *in vivo* knockout studies (Retallack et al. 2016, Meertens et al. 2017, Li et al. 2017a). The authors suggested that the virus uses another receptor to enter into the less differentiated cells. By contrast, DENV, for which AXL is also an entry factor, is dependent on this protein to infect microglia, astrocytes, and neural progenitors (Meertens et al. 2012, 2017).

### *Orthoherpesviridae*: HSV-1 and CMV

In the case of HSV-1 encephalitis, the genetic determinants in patients remain poorly documented. Data indicating that heterozygous mutations in the *SNORA31* gene found in patients could be causative of HSV-1 encephalitis were further confirmed using genetically engineered human iPSCs-derived cortical neurons and oligodendrocytes. Indeed, CRISPR-mediated deletion of *SNORA31* makes these neurons susceptible to HSV-1, similarly to neurons derived from iPSCs of patients with *SNORA31* mutations (Lafaille et al. 2019). These studies highlight the relevance of genetic manipulation of iPSCs and derived organoids to decipher key mechanisms of neuroinfection.

HSV-1 also causes microcephaly. Compared with ZIKV, HSV-1 impaired organoid size as well, and even caused their disintegration at a higher dose. However, HSV-1 and ZIKV infections induced different transcriptional responses. Additionally, while interferon (IFN) beta treatment rescued ZIKV-induced organoid alterations, it was not efficient against HSV-1 (Krenn et al. 2021). Distinct pathological features of HSV-1 were highlighted in brain organoids compared with 2D neural cultures, with acute and latency-like infections observed in both cases. The reactivation was, however, less efficient for HSV-1 in organoids, as seen in the CNS of animal models where HSV-1 reactivation is inefficient. HSV-1 was reported to spread from the organoid periphery to its inner layers (D'Aiuto et al. 2019). Modeling HSV-1 encephalitis in human brain organoids demonstrated virus-induced alteration of tissue integrity, neuronal functions, and transcriptome. Although highly reducing viral load, acyclovir used as antiviral treatment did not prevent viral damage and induction of neuroinflammation, but only did in combination with anti-inflammatory drugs, offering a potential therapeutic strategy aiming at stopping viral replication and tuning inflammatory response to mitigate subsequent brain injury (Rybak-Wolf et al. 2023).

Three studies thoroughly examined the impact of HCMV infection on brain organoids (Brown et al. 2019, Sison et al. 2019, Sun et al. 2020). HCMV was able to spread in cortical organoids and impaired their structural organization (such as neural rosettes formation) and the further generation of differentiated neurons (Brown et al. 2019, Sison et al. 2019). Similar observations were made by Sun and colleagues, who reported reduced organoid growth upon infection and alteration in the formation of cortical layers. Incubation of the virus with neutralizing antibodies restored the defects caused by infection in these settings (Sun et al. 2020).

### *Coronaviridae*: SARS-CoV-2

As expected, numerous studies used human brain organoids to assess SARS-CoV-2 neurotropism and neurovirulence, with sometimes discrepant results that may be tied to differences in multiplicity of infection used, type of protocol for organoid production, cellular composition, and age of the organoids, etc. (Ostermann and Schaal 2023). They demonstrated neurons permissiveness under these conditions and showed productive infection of neurons to different extents, and syncytia formation upon cell-cell fusion as observed in neural cultures. Evidence of neuronal death and perturbations of synaptic functions were associated with the infection, indicating a potential direct viral involvement in CNS manifestations for some authors (Bullen et al. 2020, Ramani et al. 2020, Zhang et al. 2020, Song et al. 2021, Partiot et al. 2024b). SARS-CoV-2 infection of brain organoids aberrantly rearranges synapse morphology with enlargement of presynaptic structures and increase of their number as revealed by analysis of the presynaptic marker Bassoon, and perturbs electrical activity, as shown by monitoring local field potentials of organoids cultured on microelectrode arrays. Mechanistically, infection induces an upregulation of synaptic proteins expression, the most overexpressed being Latrophilin-3 (LPHN3), a protein involved in synapse formation and maintenance. Interestingly, this protein and its receptor are also upregulated in the brain of COVID-19 cases. Swelling of synapses was dependent on LPHN3, as it could be reverted by pharmacological treatment with an agonist peptide of this protein. Treatment also reverted the altered electrical activity of infected organoids. This study also suggested a key role of microglia in synaptic pruning in this context, because adding monocytes to the cerebral organoids reverted the enlargement of synapses (Partiot et al. 2024b). The relevance of these findings with respect to natural CNS infection and COVID-19 neurological symptoms should be further addressed. Indeed, even if LPHN3 deficiency has been associated with defective electrical activity in mouse (Sando and Südhof 2021), there are no available data involving this protein in viral infections to date. Similarly, replication of SARS-CoV-2 in human iPSC-derived brain organoids induces death of cortical neurons and loss of synapses (Mesci et al. 2022).

Such a model of infection should be carefully used to address specific questions and does not allow evaluating whether the virus can actually reach and enter the brain or the CNS more globally. A choroid plexus organoid model showed that choroid plexus cells were more permissive to SARS-CoV-2 than neurons and that infection impaired the integrity and function of the blood–cerebrospinal fluid barrier (Pellegrini et al. 2020). Neuron-neuron and neuron-glia fusion, and subsequent formation of syncytia via the fusion of neurites (and more marginally soma), were reported in SARS-CoV-2-infected brain organoids (Martínez-Mármol et al. 2023).

### *Paramyxoviridae*: MeV, NiV, and HeV

Paramyxoviruses gather important neurovirulent and encephalitic viruses, including highly lethal zoonotic pathogens with pandemic potential such as HeV and NiV; however, only a few studies using brain organoids to deal with this family of viruses have been conducted to date. Infection of human brain organoids with MeV highlighted the crucial role of the viral fusion protein in neural dissemination. Notably, single mutations destabilizing the fusion protein, identified in patients with MeV encephalitis, rendered the virus highly efficient to infect and spread in organoids. A fusion inhibitory peptide was shown to prevent viral spreading (Mathieu et al. 2021). To date, no study using brain organoids for examining emerging HeV and NiV has been published, despite these viruses being highly neuroinvasive and encephalitic to animals and humans.

### *Retroviridae*: HIV-1

An elegant model of coculture between microglia and human brain organoids was used to investigate HIV-1 neuropathogenesis (Dos Reis et al. 2020). HIV-1 may cause encephalitis and targets microglia and other glial cells in the CNS, while leaving neurons uninfected (Masliah et al. 1992, Kaul and Lipton 2006). Microglia infected with HIV-1 were added to human brain organoids and were shown to attach, infiltrate, support productive infection, and produce inflammatory cytokines in the organoids, consistent with the neuroinflammatory environment seen *in vivo*. This resulted in neuronal loss and astrocytosis (Dos Reis et al. 2020). Another report using microglia-containing human brain organoids indicated that microglia were the only target cells in this model, and that they supported productive infection (Gumbs et al. 2022). This was further demonstrated in a recent model of cerebral organoids, in which hematopoietic progenitors are cocultured with human iPSCs and differentiated into microglia during organoid formation, generating organoids with a physiologically relevant percentage of microglia of ~7% (Narasipura et al. 2025).

### *Picornaviridae*: EV-A71, EV-D68, and PeV

*Picornaviridae* include notable neurotropic and encephalitic viruses, especially EV-A71, EV-D68, and parechoviruses (PeV) (Huang and Shih 2015, Leber et al. 2016, Wiley 2020, Wang et al. 2023, Liu and Long 2025). EV-A71 primarily replicates in the gut, but can invade the CNS via different means, by using retrograde axonal and trans-synaptic transport, and crossing the BBB via the infection of immune cells. It can infect peripheral nerves such as enteric and cranial nerves, and motor neurons at neuromuscular junctions (Tan et al. 2014, Lim et al. 2021, Wang et al. 2023, Gaume et al. 2024). *Ex vivo* neural models are somewhat underused to explore EV-A71 neuropathology. Tropism for motor neurons was notably demonstrated in human ESC-derived human spinal neurons (consisting of mixed cell populations, namely motor neurons, interneurons, and neural progenitor cells) and spinal cord organoids, in which neural progenitors and neurons were found infected (Chooi et al. 2024). Using both 2D and 3D models of neural, intestinal, and respiratory tissues, Tseligka and colleagues investigated intra-host adaptation by studying a variant of EV-A71 bearing a substitution in the VP1 capsid protein (Tseligka et al. 2018), acquired in an immunocompromised patient. Interestingly, this substitution was absent in the respiratory tract, but the variant was present in a mixed population in the gut. By contrast, it was present as a dominant population in the blood and cerebrospinal fluid, and was shown to be advantageous for the infection of a neural cell line, suggesting this substitution promotes neurotropism and enables neuroinvasion (Cordey et al. 2012). Mechanistically, the substitution seems to confer the virus the ability to bind heparan sulfates. However, instead of actually favoring neurotropism specifically, it rather seems to improve overall dissemination and subsequent reaching of the CNS (Tseligka et al. 2018).

In the case of EV-D68, the role of heparan sulfates in promoting virulence and neurotropism was also tackled in iPSCs-derived brain organoid. Because heparan sulfates are broadly expressed in human tissues (and also expressed in this organoid model), mutations conferring the ability to bind heparan sulfates were hypothesized to expand EV-D68 tropism (i.e. confer neurotropism). Interestingly, heparan sulfates-binding variants did not have any benefits, as all non-binding and binding variants could infect neural cells of human cerebral organoids (Sridhar et al. 2022), suggesting that gaining this ability is not or is marginally involved in the acquisition of neurotropism. Brain organoids were also used to compare a neuroinvasive and the prototypic non-neuroinvasive strain. They showed higher permissiveness to the former, as seen *in vivo* and as opposed to what is observed in the SH-SY5Y neuroblastoma cell line where both viruses replicate (Vazquez et al. 2023), highlighting the relevance of organoid models. Neuropathogenic capacity of different EV-D68 strains was effectively modeled in forebrain organoids with regard to cellular innate responses, taking advantage of cultures at different developmental stages. In less mature organoids (shorter culture period), neural stem cells, which had functional antiviral innate responses, were shown to basally express IFN-stimulated genes, priming them to respond to the infection. Accordingly, while both neuropathogenic and non-neuropathogenic strains could efficiently infect late organoids (and SH-SY5Y cells), only the neuropathogenic strain could replicate productively in early organoids, suggesting a higher permissiveness of late organoids. In addition, neuropathogenic EV-D68 counteracts IFN responses, hampering innate induction. This study further showed that the entry of both strains depends in part on sialic acid expressed in cerebral organoids (Vazquez et al. 2024). Similar findings concerning the antiviral immunity of progenitor cells were made with LACV in early forebrain organoids derived from human iPSCs, in which type I IFN-mediated innate signaling was induced within uninfected neural progenitors and limited viral spread and replication. Further, the virus antagonized this response via its specialized protein NSs (Negatu et al. 2025).

However, there are contradictory results concerning induction of and response to IFN in neural stem/progenitor cells and neurons at different maturation states, which may be linked to the different models, protocols, and viruses used, the origin of the model (human, wild-type [WT] or transgenic animal), or the type of neuronal populations studied (Farmer et al. 2013, Fantetti et al. 2016, Ferren et al. 2019, Lin et al. 2019, Winkler et al. 2019, Telikani et al. 2022, Carvajal Ibañez et al. 2023, Ferren et al. 2023, Vazquez et al. 2024, Negatu et al. 2025). Typically, while some reports indicate an association of neuronal maturation with increased susceptibility to LACV-induced apoptosis mediated by reduced IFN response, or a protection of neural stem/progenitor cells from MeV infection by IFN gamma (Fantetti et al. 2016, Winkler et al. 2019), other data show that human neuronal differentiation induces the upregulation of type I IFN pathway and increase of functional IFN response, and suggest that immature neurons and progenitors are more susceptible to alphaviruses infection, or that adult/more differentiated neurons become non-permissive to infection in the case of MeV for example (Dhondt et al. 2013, Farmer et al. 2013, Ferren et al. 2019, Welsch et al. 2019, Ferren et al. 2023). This aspect therefore warrants more precise characterization and use of pertinent systems with respect to human neuropathology.

EV-D68 tropism remains elusive during human infection; however, *in vivo* and *in vitro* studies indicate that it targets spinal cord motor neurons, cortical neurons, and astrocytes (Brown et al. 2018, Poelaert et al. 2023). By infecting iPSCs-derived spinal cord organoids containing spinal motor neurons, interneurons, and glial cells, it was shown that historic strains of EV-D68 were unable to replicate, contrary to contemporary neuropathogenic strains, even infecting deep inside the organoids, with the extracellular release of progeny virions but absence of obvious cytopathic effects (Aguglia et al. 2023).

In the same family, certain PeV such as PeV-A3 can cause CNS disease, while others do not, such as PeV-A1. In human brain organoids, both viruses are able to infect and replicate in astrocytes and neurons (Capendale et al. 2024). However, PeV-A1 infection was more efficient than that of PeV-A3, contrary to observations made in neuroblastoma cell line (Westerhuis et al. 2012, Capendale et al. 2024). The major difference between neuropathogenic and non-neuropathogenic PeV was a strongly increased inflammatory response upon PeV-A3 infection of organoids, correlating clinical data, suggesting that neuropathology is mediated by neuroinflammation induction, rather than viral replication itself (Koyuncu et al. 2013, Capendale et al. 2024).

### *Matonaviridae*: RuV

As a last example, rubella virus (RuV) infection during pregnancy can result in neurological pathology, but the viral tropism and mechanisms of pathogenesis in the CNS are still poorly defined. To address these questions, brain organoids were used to model the early developing brain and engrafted with fetal primary human microglia. This model highlighted the strong viral tropism towards microglia, revealed by capsid staining in these cells, but not in other neural cells (Popova et al. 2023), similarly to HIV-1 and ZIKV congenital infection, but opposite to other pathogens such as HSV or HCMV (Rock et al. 2004, Retallack et al. 2016, Lum et al. 2017). Microglia infection was shown to require diffusible factors from non-microglial cells in order to occur, and infection overall impacted the expression of genes involved in cerebral development (Popova et al. 2023).

### Drawbacks of the model

One of the reported limitations of most brain organoid models is the lack of the resident macrophages of the CNS, microglia, because they derive from the mesoderm, contrary to the other neural cells originating from the neuroectodermal lineage (Barreras et al. 2023). However, protocols to produce microglia-containing brain organoids are being developed by adding immortalized/primary microglia or macrophage progenitor to previously generated organoids (Abreu et al. 2018, Dos Reis et al. 2020, Xu et al. 2021). Besides, it was actually reported that cells with typical features of microglia (morphology, phenotype, functions) can intrinsically develop in human brain organoids, disproving the consensus to date (Ormel et al. 2018, Gumbs et al. 2022). In the first organoid models, the presence of oligodendrocytes was also somehow lacking, but protocols to induce oligodendrogenesis and myelination in brain organoids were reported in recent years (Madhavan et al. 2018, Shaker et al. 2021). Just like neural cultures, most organoids lack internal vasculature, BBB, choroid plexus, and immune system. In addition, organoids resemble more a embryonic brain structure than that of an adult, and the protocols for organoid generation are quite heterogeneous, which can make comparisons difficult between studies. The technique is still not standardized and homogenous between labs and its variability is thus relatively high. In most cases, size and shape of the organoids are not controlled parameters, although size- and shape-controlled organoids are being developed (Pamies et al. 2017). Size is a critical factor, because as they mature and grow until reaching a certain limit, necrosis in the inner core of the organoids occurs as a result of limited diffusion and subsequent depletion of oxygen and nutrients. This leads to additional variability that can still be compensated by increasing the number of samples per condition (Grebenyuk and Ranga 2019). From a practical point of view, infection, treatment or analysis (such as monitoring of viral spread) of organoids can be somewhat tricky due to their 3D structure (Table 1).

## A window into the brain: organotypic brain cultures

Developed several decades ago (Humpel 2015), organotypic brain cultures (OBCs) are an even more complex and physiological model, inasmuch as they represent an open window directly on the brain. They are less used than primary neural cultures because of the higher complexity to prepare them. Additionally, they are less easy to implement but have shown their great value for studying neurotropic viruses in the last 20 years. Historically, organotypic culture of nervous tissue was notably initiated in 1962 with the work of Bousquet and Meunier on rat hypophysis fragments (Bousquet and Meunier 1962), further to which two main methods were sequentially developed. The first one is the roller-tube technique, where organotypic slices are cultured on flat-sided culture tubes under slow rotation with a low amount of medium, allowing regular alternation of feeding and aeration (Gähwiler 1981, Gähwiler et al. 1997). Optimization of this technique in the 1990s led to the membrane interface culture method, where slices are kept on semipermeable membranes (Fig. 2) (Stoppini et al. 1991, Gähwiler et al. 1997, Daviaud et al. 2013).

OBCs consist of slices of brain substructures (cerebellum, hippocampus, brainstem, olfactory bulb, etc.) typically prepared with a tissue chopper (in the open air) or a vibratome (in liquid medium). For the tissue chopper protocol, these cultures are then maintained on a semiporous membrane such as they reside on an air–liquid interface, receiving nutrients from the medium underneath the membrane and oxygen from the upper side, or in liquid medium for the vibratome protocol (Humpel 2015, Welsch et al. 2017). A standardized tissue chopper protocol for virology was notably established by our lab a few years ago (Welsch et al. 2017). OBCs have several notable advantages. They retain the original cytoarchitecture and all the neural cell types, and can be obtained from virtually any animal model (rodents, ferret, dog, primate, etc.) or even humans (including post-mortem or from surgical resection). They are ethically preferable over *in vivo* experiments because several slices from several substructures can be prepared from one animal, thereby enabling the testing of several conditions and reducing animal toll. In addition, euthanasia is quick, thereby reducing animal pain because no animal manipulation is required.

### *Bornaviridae*: BoDV-1

These *ex vivo* cultures offer an unique opportunity to investigate early events of CNS viral infection. Examination of BoDV-1 pathogenesis in newborn rat hippocampal slice cultures revealed a selective neuronal loss of dentate granule cells upon infection, as observed *in vivo* in infected newborn rats, whereas BoDV-1 is non-cytolytic in primary neuron cultures, indicating that these *ex vivo* cultures are relevant to model BoDV-1 neuropathogenesis (Mayer et al. 2005). OBCs can also be used to compare different substructures in terms of their intrinsic response to infection and to factors like cytokines in the absence of a circulating immune system. For BoDV-1, murine organotypic cerebellar and hippocampal cultures supported viral proliferation with neurons being the main infection target, as shown by immunofluorescence labeling. Additionally, treatment with IFN gamma was fully efficient for inhibiting BoDV-1 infection in cerebellar slices, seemingly by putting non-infected neural cells in an antiviral state and preventing infection, contrary to hippocampal slices for which efficiency was lessened. This observation highlights a differential sensitivity to this cytokine depending on the brain region (Friedl et al. 2004).

### *Orthoherpesviridae*: HSV-1 and CMV

OBCs are particularly suitable to study initial viral neurotropism, both at the cellular and brain region scales, as was done for HSV-1 in neonate mouse and rat OBCs for example (Braun et al. 2006, Cohen et al. 2011). HSV-1 neurotropism was specifically oriented towards leptomeningeal, cortical, periventricular, and hippocampal areas, infecting meningeal, ependymal, and undifferentiated cells, but only a few differentiated astrocytes or neurons, paralleling observations made in intracerebrally inoculated animals (Braun et al. 2006). Age of the animal at the time of slicing is an important factor for infection susceptibility, as shown in this study where HSV-1 infection of adult OBCs was far less extensive than that of neonate animals (Braun et al. 2006), seemingly because of a different maturity state of the neural cells and cellular intrinsic innate response. Similar observations were made with murine CMV (MCMV) in murine OBCs (Kawasaki et al. 2002, van den Pol et al. 2002). Recently, human fetal OBC was developed to investigate HSV infection in a more relevant system and demonstrated a consistent cell tropism (towards astrocytes and neurons) and neuropathology compared with what is observed in the brain of neonatal and adult cases of HSV encephalitis, and notably virus-induced necroptosis (Young et al. 1965, DeBiasi et al. 2002, Wnęk et al. 2016, Rashidi et al. 2024). Such findings should, however, be treated cautiously because fetal brain tissues differ from neonatal ones, in that they represent distinct developmental and thus maturity stages. Conversely, this model would be relevant for viral infections occurring during pregnancy such as ZIKV infection.

### *Paramyxoviridae*: MeV, NiV, and CDV

A large corpus of data regarding Paramyxovirus encephalitis has been gathered thanks to OBCs. In particular, the initial cellular targets of MeV infection in human CNS remained elusive, and several studies addressed this knowledge gap. MeV infection of organotypic hippocampal cultures from IFN signaling-deficient (IFNAR^KO^) mice expressing MeV receptor showed increased viral replication compared with immunocompetent cultures, and that all neural cell types were permissive to MeV infection in both settings. However, astrocytes and microglia became refractory to infection over time in culture in immunocompetent slices, but not in IFNAR^KO^ slices. This was concomitant to the development of an astrogliosis phenomenon induced by the slicing procedure that depends on IFN signaling, presumably putting responder cells (astrocytes and microglia) in an antiviral state while leaving neurons and, to a lower extent, oligodendrocytes permissive to the infection (Welsch et al. 2019).

This model describing the role of IFN signaling in the control of astrogliosis and of MeV CNS infection and early permissiveness of glial cells, and more generally, MeV initial neural tropism, has been recently investigated further in hamster organotypic cerebellar cultures (Ferren et al. 2023). Hamster is a more relevant model for MeV because hamster brain is naturally susceptible to infection contrary to mouse, harboring a too strong type I IFN response as observed elsewhere for NiV (Dhondt et al. 2013). This study notably demonstrated that WT virus and a neuroinvasive hyperfusogenic variant (bearing a single substitution found in MeV encephalitis patients in the fusion protein) were able to infect all CNS cell types. The WT virus infected few cells in total and showed limited spreading compared with the neuroinvasive variant. As observed in MeV encephalitis patients, the initial tropism of the neuroinvasive variant was strongly skewed towards neurons. Strikingly, stimulation with type I IFN strongly impaired astrocytes and microglia permissiveness to both WT and mutant viruses, leaving neurons as almost the only infected cells. Infection after 7 days of culture, that is, after the development of IFN-dependent astrogliosis, led to the same observation (Ferren et al. 2023). Incidentally, in murine OBCs this time, this MeV variant strongly induces IFN-stimulated genes (Mathieu et al. 2021). Interestingly, astrogliosis is a feature of MeV encephalitis and astrogliosis-related loss of permissiveness of astrocytes and microglia could thus explain why infection of these cells is barely detectable in post-mortem patient samples (Allen et al. 1996, McQuaid and Cosby 2002, Ferren et al. 2019). In this case, OBCs would mimic MeV encephalitis progression from initial cellular targets to terminally infected cells at disease outcome (early and late tropism, respectively).

MeV and CDV early tropism and neurovirulence were compared in olfactory bulb, hippocampal and cortical organotypic cultures from naturally susceptible hosts (non-human primate, dog, and ferret). This study showed similar infection levels and initial tropism between these closely related viruses, with predominant infection of microglia and neurons (Laksono et al. 2021). In addition, the ability of exogenous T cells to migrate to and clear neurotropic viruses from infected neuron, and the role of IFN gamma in this non-cytolytic viral clearance, have been highlighted in MeV- and WNV-infected murine hippocampal cultures (Stubblefield Park et al. 2011). Addressing viral spread in the CNS is particularly relevant in OBCs. In MeV-infected hippocampal cultures, viral spread occurred unidirectionally, in a retrograde fashion. It seemed to be mainly cell-cell contact-dependent but independent of the release of infectious units, suggesting the involvement of microfusion events at synaptic contacts (Ehrengruber et al. 2002).

### *Flaviviridae*: ZIKV and WNV

Neurotropism of arboviruses, such as ZIKV and WNV, and effects of infection on CNS physiology, were also tackled in OBCs. In murine OBCs of different embryonic developmental stages, ZIKV was found to target preferentially neocortical proliferative and developing midbrain areas, and to impair neuronal migration. Intriguingly, apoptosis of uninfected cells was observed, potentially limiting viral dissemination (Rosenfeld et al. 2017). Combined use of cerebellar organotypic cultures and brain organoids revealed that ZIKV-infected monocytes had increased adhesion and transmigration ability and promoted infection and viral dissemination within the neural cultures (Ayala-Nunez et al. 2019).

Neuronal apoptosis is also a hallmark of WNV neuropathogenesis, and was suggested to be mediated by death receptor signaling in infected neurons in murine OBCs. Signaling of death receptors, cell surface receptors that are members of the tumor necrosis factor receptors family and whose binding triggers apoptosis notably via the activation of caspases, is upregulated in WNV-infected OBCs, and inhibition of Caspase-8 minimizes virus-induced tissue injury (Clarke et al. 2014). Neurons and astrocytes are found infected in this model, but not microglia, which instead appear more to phagocyte debris from infected cells. As observed in MeV studies, data also point out the role of microglia and astrocyte activation upon WNV infection, which influences WNV pathogenesis (Clarke et al. 2014, Stonedahl et al. 2022). Indeed, depletion of microglia in OBCs promotes viral growth and cell death, suggesting a role in limiting WNV dissemination in the CNS likely via phagocytosis of viral particles or infected neural cells (Stonedahl et al. 2022).

### *Coronaviridae*: SARS-CoV-2

Key aspects of SARS-CoV-2 neuropathogenesis were effectively modeled in OBCs, which turned out to be a choice model to rapidly gather knowledge at the beginning of the pandemic about this rapidly emerged pathogen. It could infect and disseminate in hamster brainstem and cerebellar organotypic cultures by targeting mainly granular and Golgi neurons, and induce apoptotic, necroptotic, and pyroptotic signatures (Ferren et al. 2021), consistent with *in vivo* data involving infection-induced brainstem neuropathology (Bulfamante et al. 2021, Coleon et al. 2024). Other reports mainly indicate infection of astrocytes, and limited or undetectable infection of neurons in human cortical OBCs and hamster cerebellar cultures (Andrews et al. 2022, Lamoureux et al. 2022). Such discrepancies could be tied to differences in cellular subpopulations between the OBCs used, in culture conditions, in viral doses used for infection, in the method of infection or in the virus variant used. Interestingly, data obtained in post-mortem human OBCs and neuronal cultures indicate that retention of SARS-CoV-2 particles in synapses is correlated with impairment of synaptic organization and functions. Indeed, as observed in brain organoids, infection increases presynaptic contents and affects synapse organization. Synaptic dysfunction appears thus to be the result of synapse reorganization and trans-synaptic accumulation of viral particles leading to local hindrance, which may contribute to neurological disorders observed in patients (Partiot et al. 2024b).

### *Retroviridae*: HIV-1

A model of human OBC from healthy surgical resection of adult brain tissue was recently developed to study HIV-associated neuropathology, and was shown to be almost fully viable for up to 4 weeks in culture as measured by cell dissociation and flow cytometry. Using patient-matched T cells exposed to HIV-1 and cocultured with these human OBCs, mimicking the Trojan horse mechanism of neuroinvasion, this study demonstrated efficient infection (of astrocytes and myeloid cells notably), which spread in the slices without impairing viability. This model has great potential to relevantly investigate mechanisms of HIV neuroinfection, and evaluate antiretrovirals and neuroprotective treatments (Van Duyne et al. 2024).

### Drawbacks of the model

OBCs are useful tools to establish proof-of-concept and for screening antiviral molecules, and have the advantage of being more predictive of the *in vivo* efficiency and toxicity of the tested drugs than 2D cell cultures. This was shown, for instance, for MeV with fusion inhibitory peptides and inhibitors of host factors needed for infection, for a tick-borne encephalitis virus with antiviral small interfering RNA, or for SARS-CoV-2 and the ineffectiveness of remdesivir and hydroxychloroquine (Maffioli et al. 2012, Welsch et al. 2013, 2017, Bloyet et al. 2016, Ferren et al. 2021). However, like organoids, OBCs lack vascular and circulating immune systems. As mentioned above, development of astrogliosis over time renders OBCs less susceptible to infection, and similarly, the susceptibility of OBCs from aged animals is generally decreased compared with neonate or young animals (Humpel 2015, Welsch et al. 2017, 2019, Ferren et al. 2023). Instead of using OBCs before the onset of astrogliosis, it is also possible to culture them for ~2 weeks until astrogliosis has ceased before performing infection experiments, however, taking into account the tissue remodeling due to the healing process (Humpel 2015) (Table 1).

## Contribution and relevance of *ex vivo* models for the understanding of neurotropic pathogens

As previously mentioned, *ex vivo* systems are useful tools to address basic questions relevant for all families of CNS-targeting viruses, and allow pertinent mimicking of the *in vivo* context (Table 2). Common study questions typically include viral tropism that is readily investigated in these systems, which allow the comparison of closely related viruses, as illustrated by the study of CDV and MeV, or ZIKV and DENV. They also allow to easily assess the antiviral activity of candidate molecules. These are general questions that can be tackled for every virus, however, others are specific for some viral properties, for example, related to acute infection, cytopathic effect, viral adaptation, or evolution within the tissue. As a matter of fact, neural *ex vivo* models were differently used depending on the virus families and orders. Indeed, for Rhabdoviruses and RABV in particular, which initially infects motor neurons at neuromuscular junctions, or HSV, for which infection starts from mucosal epithelium where the virus enters the nervous system via axon termini of sensory neurons (Ludlow et al. 2016), these models have been pivotal to understand retrograde axonal transport of these pathogens and access to the CNS. On the contrary, very few studies have investigated, for example, how Paramyxoviruses spread within and between neural cells and, notably, how their ribonucleoprotein complexes are transported within these cells. On the contrary, questions related to the involvement of the paramyxoviral fusion machinery (the surface glycoproteins allowing viral entry into target cells) in the neural spread have been widely addressed *ex vivo*, leading to the conclusion that mutations destabilizing the fusion machinery facilitate viral spread in CNS tissues. For Bornaviruses, *ex vivo* CNS models were particularly used to study the cytopathic effects of these viruses and the role of their virulence factor. Concerning the *ex vivo* study of Retroviruses and HIV in particular, the immunological features of the CNS infection are especially examined, with a focus on the interaction between the pathogen and microglia. Co-infection studies would also be of interest, because opportunistic pathogens can sometimes infect the CNS of AIDS patients. For instance, measles inclusion-body encephalitis is a type of encephalitis caused by MeV occurring in immunosuppressed patients because of HIV infection, notably. Nobody addressed the possible interconnexion between measles and HSV or HTLV-1 in infected patients knowing that this virus can also spread to the CNS and lead to encephalitis and HTLV-1-associated myelopathy/tropical spastic paresis, an aggressive neurodegenerative disease (Rocamonde et al. 2023a,b). The main CNS-targeting pathogen in AIDS patients was HCMV before the democratization of antiretroviral therapy (Ludlow et al. 2016).

**Table 2.** Selected examples of key findings made in *ex vivo* neural models and their corresponding *in vivo* relevance with regard to neuroinvasive and neurotropic viruses.

Virus	Key insights in *ex vivo* models	Corresponding *in vivo* relevance	References
BoDV-1	Neural cultures: neuronal loss due to viral interference with neurogenesis	Neuronal death upon infection of neonatal animals	Weissenböck et al. 2000, Hans et al. 2004, Brnic et al. 2012, Scordel et al. 2015
	Organotypic cultures: loss of hippocampal dentate granule cells upon infection		Mayer et al. 2005
	Neural cultures: neuroprotective role of the viral X protein, which inhibits apoptosis induction in neurons	Neuroprotective role of the viral X protein in a mouse model of amyotrophic lateral sclerosis	Szelechowski et al. 2014, Tournezy et al. 2024
ZIKV	Organoids: reduction of organoid size, disorganization, apoptosis, and loss of neural progenitors induced by infection	Brain development defects and microcephaly upon fetal infection	Garcez et al. 2016, Qian et al. 2016, Li et al. 2017b
	Neural cultures and organoids: AXL-independent viral entry into neural progenitors and neurons	*Axl* knockout in neonatal mouse does not prevent brain infection	Wells et al. 2016, Li et al. 2017a
HSV-1	Organoids: reduction of organoid size induced by infection, destruction at high infection dose	Brain development defects and microcephaly upon fetal infection	Krenn et al. 2021
	Organotypic cultures: viral tropism targeting neurons and astrocytes, and virus-induced necroptosis in fetal OBC	Tropism oriented towards neurons and astrocytes, and induction of apoptosis/necroptosis in the brain of HSV encephalitis cases	Young et al. 1965, DeBiasi et al. 2002, Wnęk et al. 2016, Rashidi et al. 2024
NiV	Neural cultures: efficient viral replication in primary olfactory neurons and cytopathic effect	Neuroinvasion via the olfactory entry route in the hamster model	Munster et al. 2012, Borisevich et al. 2017, Ozdener et al. 2023
	Organotypic cultures: high susceptibility of the choroid plexus in infected murine OBC	High susceptibility of the cells of the ventricular system in ferrets and pigs	Weingartl et al. 2005, Clayton et al. 2012, Gellhorn Serra et al. 2024
MeV	Organoids and organotypic cultures: hyperfusogenicity and preferential neuron tropism of variants with mutated fusion protein, linked to IFN pressure and astrogliosis	Neurovirulence of variants with mutated fusion protein in brain of patient with encephalitis, strong tropism for neurons, and occurrence of astrogliosis	Allen et al. 1996, McQuaid and Cosby 2002, Ferren et al. 2019; Welsch et al. 2019, Mathieu et al. 2021, Ferren et al. 2023
SARS-CoV-2	Organoids and organotypic cultures: upregulation of LPHN3 and LPHN3-dependent aberrant enlargement of synapses upon infection	Upregulation of LPHN3 and its receptor in the brain of COVID-19 patients	Partiot et al. 2024b
	Organotypic cultures: viral expression levels upon infection comparable to that of naturally infected human brain samples, suggesting physiological replication levels	Viral RNAs detectable at relatively low levels in the cortical temporal lobe of patients who died of COVID-19	
	Organotypic cultures: productive infection of the brainstem with preferential tropism for granule neurons in motor and sensory areas of the tissue	Neuroinvasion via the vagus nerve, and brainstem productive infection characterized by neurodegenerative features tied to neurological disorders	Bulfamante et al. 2021, Ferren et al. 2021, de Melo et al. 2023, Woo et al. 2023, Andersson and Tracey 2024, Coleon et al. 2024
HIV-1	Organoids: productive infection of microglia, leading to production of inflammatory cytokines and subsequent neuronal loss and astrocytosis	Tropism oriented towards microglia and neurons left uninfected in encephalitis cases, neuroinflammation	Masliah et al. 1992, Kaul and Lipton 2006, Dos Reis et al. 2020, Gumbs et al. 2022, Narasipura et al. 2025

The knowledge gap is even more pronounced for viruses requiring high/maximum level of containment (biosafety level [BSL-]3 and 4). BSL-4 pathogens notably include the highly neurotropic Henipaviruses HeV and NiV, zoonotic viruses causing severe respiratory syndromes and encephalitis in humans with case fatality rates that can be >90% depending on the outbreaks (Li et al. 2023). In culture, HeV and NiV induce the formation of syncytia (multinucleated cells) and strong cytopathic effect in most cell types (Eaton et al. 2006). The emerging NiV is highly efficient at entering and invading hamster brainstem and cerebellar organotypic cultures (Ferren et al. 2021), but more studies are needed to explore neuroinvasion and decipher neuropathogenesis of Henipaviruses in OBCs. Indeed, the way by which HeV and NiV can reach the CNS, the way they spread within the nervous tissue, or what neural cells are primarily targeted in the early stages of infection, are still elusive, because the work done in the BSL-2 context for other Paramyxoviruses like MeV or CDV is more difficult to accomplish for BSL-4 viruses. NiV is highly neuroinvasive, neurotropic, and neurovirulent, as shown by numerous studies performed in 2D models, but only a few in more relevant 3D *ex vivo* models (Ferren et al. 2021, Gellhorn Serra et al. 2024). In the study of Ferren and colleagues, NiV infection of brainstem and cerebellar slices was used as a comparison for SARS-CoV-2 infection in these models. NiV targeted different tissue areas and spread more widely compared with SARS-CoV-2 (Ferren et al. 2021). Murine OBCs were also used to decipher NiV neurotropism. The virus replicated in these cultures, but without detectable release of infectious viral particles, suggesting cell-to-cell spread (Gellhorn Serra et al. 2024). Of note, the choroid plexus was seemingly found to be strongly infected, in accordance with *in vivo* data indicating infection of the ventricular system (Weingartl et al. 2005, Clayton et al. 2012). This observation supports the hypothesis that NiV could enter the CNS by infecting the cells of the blood–cerebrospinal fluid barrier (Gellhorn Serra et al. 2024). However, in this study, slices were not standardized in terms of size and quality of the explants, and infection was performed 4 days after isolation, which may not be very pertinent because of the induction of astrogliosis over the time in culture (Welsch et al. 2017, 2019, Ferren et al. 2023). Moreover, mouse is less relevant than, for example, hamster, to study Henipaviruses, limiting the interpretations of the results (Wong et al. 2003, Juelich et al. 2023). We and others are currently investigating infection by NiV and HeV in relevant *ex vivo* systems (OBCs and organoids), notably with respect to neurotropism and characterization and comparison of the fusion machineries of the different strains of Henipaviruses, or evaluation of antiviral strategies (Ferren 2021).

Interestingly, these viruses can cause acute, but also relapsed and late-onset encephalitis, up to several years following initial infection (Eaton et al. 2006). In the BSL-4 setting, acute infection, cytopathic effect, and viral evolution/adaptation are generally studied over a few days in both *in cellula* and *ex vivo* models. On the contrary, long-term (at the scale of several months or years) *ex vivo* studies are not achievable, and it is thus not possible to model late encephalitis so far and to decipher how the virus stays “inactive” in the CNS for a long time. More globally, high biosafety levels technically limit the feasibility of the studies, with for instance the need to inactivate the samples with harsh and strict protocols to analyze them outside of BSL-4 laboratories. Alternatively, *ex vivo* systems could be combined with the use of virus-like particles, allowing working in BSL-2 conditions.

The questions addressed and further interpretations also depend on the properties of the model used (species and region of the CNS from which it originates, developmental stage, etc.), notably for OBCs. This is typically illustrated with a study comparing the neurovirulence of ZIKV, WNV, and the Usutu virus (another closely related neurotropic arbovirus, which co-circulates with WNV in Europe) in human fetal OBCs (Marshall et al. 2024), and a study investigating HSV infection in human fetal OBCs (Rashidi et al. 2024). While this model is particularly relevant for infections occurring during pregnancy (e.g. in the case of ZIKV), it is less true for infections occurring in neonates and adults (in the case of HSV and WNV), because fetal, neonatal, and adult tissues differ significantly from each other. For Herpesviruses, specific questions related to latency and reactivation, and the link with the immune system, have been tackled *ex vivo*, with better relevance compared with classical animal culture models. Indeed, CNS neurons derived from human iPSCs are permissive to HSV-1 (acute infection), and can also exhibit features of viral latency similar to those observed in animal models. In human iPSCs-derived brain organoids, acute infection with evidence of viral trafficking from the outer surface to inner layers, as well as latency, could also be observed. Reactivation could be induced in both models but with less success in organoids, mirroring the low reactivation efficiency *in vivo* (D'Aiuto et al. 2019).

## Neuronal infection in peripheral organs: interplay with the CNS and the innate immunity

Neuroinvasiveness, neurotropism, and neurovirulence of the aforementioned viruses also manifest in peripheral organs, but quite a few studies make use of *ex vivo* systems to investigate infection of peripheral neurons, notably. Numerous viruses target neural cells in peripheral organs before eventually reaching the well-protected CNS. In this regard, the enteric nervous system, containing >500 million neurons, can impact the CNS and be a portal of entry upon infection of enteric neurons, highlighting the increasingly studied gut-brain axis (Valdetaro et al. 2023). Peripheral infection and inflammation can also affect the CNS, for instance inducing sensitization and alterations of the BBB, microglial activation, and parenchymal inflammation (Varatharaj and Galea 2017). It is also noteworthy that while some organs such as the liver only receive innervations, others like the lung contain intrinsic neurons (Delalande et al. 2004, Freem et al. 2010, Bower et al. 2014).

Peripheral neurons are notably targeted by HSV-1, SARS-CoV-2, human coronavirus OC43 (HCoV-OC43), varicella-zoster virus (VZV), or RABV. Infection of peripheral neurons is almost never investigated in *ex vivo* models of peripheral organs. The attention is probably more focused on the most obvious and abundant cells present in these tissues, such as enterocytes and other epithelial cells in the gut, hepatocytes in the liver, or pneumocytes in the lung. In addition, most peripheral organs possess only nerve endings, which may be difficult to maintain for a long time in culture. Consequently, most data come from *in vivo* studies. HSV-1 was shown to infect and persist in enteric neurons in a mouse model, inducing the recruitment of macrophages and T cells, ultimately leading to gastrointestinal inflammation and neuromuscular dysfunction (Brun et al. 2018, 2021). Similarly, infection of mice with WNV results in enteric neurons and enteric glial cells damage and loss, and subsequent gut dysmotility, via T cells infiltration (Janova et al. 2024). VZV, which can also cause encephalitis, is able to infect dorsal root ganglia and enteric neurons of guinea pigs infected intradermally (Chen et al. 2011).

In COVID-19 cases, SARS-CoV-2 antigens were detected in neurons of the myenteric plexus, which innervates the muscular layers of the gut and is responsible for peristalsis (Gray‐Rodriguez et al. 2022). In this regard, enteric neurons may be a possible entry into the CNS for SARS-CoV-2, notably given that these neurons are trans-synaptically connected to CNS neurons, although this assumption requires to be thoroughly tested (Valdetaro et al. 2023). Similarly, the virus may also use sensory neurons in the lung to travel retrogradely towards the CNS, notably via the vagus nerve (Yavarpour-Bali and Ghasemi-Kasman 2020, Bulfamante et al. 2021, Ferren et al. 2021, Woo et al. 2023, Andersson and Tracey 2024). The olfactory epithelium was confirmed as a major entry route into the brain of hamster infected with SARS-CoV-2, which was shown to perform retrograde and anterograde axonal transport in neuron-epithelial *in vitro* microfluidic models, but nerve terminals of the orofacial mucosa, for example, are also a possible CNS entry point (Fenrich et al. 2020, de Melo et al. 2023). A similar mechanism could be at play for the enteric nervous system. By comparison, HCoV-OC43 travels trans-synaptically along axons in neuronal cultures (Dubé et al. 2018), TBEV uses autonomic nerves of the enteric nervous system plexus to reach the CNS in mouse (Nagata et al. 2015), and EV-A71 is strongly suggested to travel retrogradely in axons of peripheral autonomic nerves to the CNS (Li et al. 2019).

Although this is still poorly characterized, gut infection can be a cause of neuroinflammation and, as such, viruses can induce neuropathology at a distance, without necessarily directly infecting neurons. As an example, exposure of rat gut to HIV-1 Tat protein leads to activation of enteric glial cells, and this neuroinflammation also propagates to the CNS via cell-to-cell signaling in the glial network (Esposito et al. 2017). The gut-brain axis is increasingly studied, including in virology, and in this context, gut infection and inflammation are proposed or reported to affect the BBB and the CNS at a distance. Besides infecting peripheral neurons to eventually enter the CNS, viruses infecting the gut can cause an inflammatory response that may, for example, sensitize and permeabilize the BBB (through alteration of the integrity of tight junctions, induction of endothelial dysfunction and astrocytic damage), facilitating entry of inflammatory factors or virus into the brain, inducing a neuroinflammation (Varatharaj and Galea 2017, Valdetaro et al. 2023, Yata 2024). BBB disruption is demonstrated in acute COVID-19 cases, but also in patients with long COVID-associated neurological sequelae, linked to sustained and systemic inflammation (Valdetaro et al. 2023, Greene et al. 2024).

Other peripheral neurons can modulate host immune and inflammatory responses upon infection. Peripheral tissues and organs are innervated by sensory neurons, which are the target for several viruses. Some sensory neurons can signal to immune cells via the release of neuropeptides, cytokines, and other molecules (Saraiva-Santos et al. 2024). In the lung, for instance, sensory neurons were shown to exert anti-inflammatory responses (Tamari et al. 2024), and in the case of influenza, an airway-to-brainstem sensory pathway was shown to mediate onset of sickness behavior and response to infection (Bin et al. 2023). HSV and VZV infect sensory neurons and establish latency in sensory ganglia. From peripheral nerve fibers, they use retrograde axonal transport to reach the cell bodies in sensory ganglia, and anterograde transport to the skin and mucosa to spread upon reactivation (Koyuncu et al. 2013). Dorsal root ganglia *ex vivo* and sensory neuron cultures were shown to be susceptible to HSV-1 infection, and demonstrated that viral attachment and entry rely on heparan sulfates expressed at the surface of sensory fibers (Sharthiya et al. 2017). Regarding antiviral responses to HSV-1, compartmentalized cultures of murine sensory neurons from trigeminal ganglia showed that these cells respond to IFN beta by axon terminals and soma, enabling control of the infection (Rosato and Leib 2015). Both the neurons and the virus implement complex strategies to antagonize the other party. For instance, the neuropeptide calcitonin gene-related peptide (CGRP) is secreted by sensory neurons and protects Langerhans cells against HSV infection by downregulating viral entry receptors (Cohen et al. 2022), but conversely HSV-1 inhibits CGRP expression of rat primary trigeminal neurons (Hamza et al. 2007). More recently, sensory and autonomic neurons were shown to be susceptible to SARS-CoV-2 infection *in vivo* and in primary neuronal cultures. The virus was detected in trigeminal and dorsal root ganglia (sensory ganglia) and superior cervical ganglia (autonomic ganglia). The rapid invasion of the peripheral nervous system (PNS) and CNS before viremia, and the strongest detection of the virus in functionally connected brain regions (e.g. brainstem for the trigeminal ganglia projections), led the authors to postulate an alternative entry route into the brain besides the olfactory one (Joyce et al. 2024).

As previously detailed, numerous studies rely on *in vivo* animal experimentations or post-mortem investigations (notably immunostainings), which are not necessarily representative of the initial steps of infection, virus repercussions on the BBB and the CNS (neuropathology at a distance), and viral spread towards the CNS. While primary peripheral neuron cultures and ganglia explants are to some extent used to study viral neuroinfection, more complex *ex vivo* PNS models, such as ganglia organotypic cultures, PNS organoids, PNS-CNS assembloids (Rockel et al. 2023, Koyanagi et al. 2024), or peripheral organoids containing innervation, are lacking in this line of research. Likewise, gut *ex vivo* systems are not commonly used to investigate the infection of peripheral neurons (Barreto-Duran et al. 2024, Lulla and Sridhar 2024, Yata 2024), although models containing gut plexuses could be (and are already to some extent in the form of gut slices kept on culture inserts, for instance) implemented, provided that the viability and renewal/proliferation of epithelial cells are better managed (Schwerdtfeger et al. 2016, Biel et al. 2022, Jung and Kim 2022). Oher *ex vivo* models of organs of interest for the study of neurotropic virus, in particular organotypic cultures of lung (Nicholas et al. 2015, Ferren et al. 2021) or liver for example (Ogire et al. 2024, Lalande et al. 2025), are in their infancy. Of note, a study reports the culture of murine embryo lung explants containing intrinsic neurons (Bower et al. 2014). In this sense, examining and monitoring neuronal infection in *ex vivo* models of peripheral organs, whose development and use are still rather late in virology (Lalande et al. 2025)—although mainly neuronal termini might still be present (probably for a few hours/days) in the tissues in the case of organotypic slice cultures—and even combining them with CNS *ex vivo* models, could be pivotal in understanding neuropathogenesis. Alternatively, explants could be prepared from animals infected *in vivo*, and used to address basic questions such as viral tropism or dissemination from non-neuronal cells to nerves.

## Next-generation *ex vivo* model technologies

Overall, organoids are a new technology still in development that needs to be further characterized, and their use for the study of neuroinfectious viruses is in its infancy. Further improvements will likely include the use of assembloids (combined organoids that could be used to study viral spread among brain regions, for example), transplantation of human organoids in an immunodeficient animal model to enable viral infection of human neural cells in vascularized and more mature organoids, and brain- and organoid-on-chip technologies (Pașca 2018, 2019, Castiglione et al. 2022, Fan et al. 2022, Widerspick et al. 2023). Usually of higher complexity compared with organoids, organ-on-chip and more specifically brain-on-chip have been developed in recent years and their use in virology research is still in its early stages. Several designs exist but they basically consist of different cell types of the CNS (or brain organoid) in a compartmentalized microfluidic chip including biosensors that enables the biomimicry of the brain, recapitulating cell interactions, tissue/organ functions, biophysical forces, while allowing real-time monitoring and assessment of multiple parameters (Tang et al. 2020, Shahabipour et al. 2023). These 3D engineered microfluidic devices reconstitute several physiological aspects and functions of the organ. The few studies using this technology for neurotropic viruses focused, for example, on pseudorabies virus spread between cells and transport in axons (Liu et al. 2008, Johnson et al. 2016, Tang et al. 2020), or SARS-CoV-2 impact on BBB and neuroinflammation in a combined lung-brain chip model (Wang et al. 2024). Similarly, there are very limited examples of genetic editing of cerebral *ex vivo* models to study viral infections, compared with the study of neurodegenerative diseases or cancer (Lafaille et al. 2019, Hendriks et al. 2020, Nassor et al. 2020, Zhou et al. 2021, Meier et al. 2025, Pagliaro et al. 2025). An interesting case of genetic engineering of organoids to investigate virus–host interactions is the establishment of a gene knockout biobank of intestinal and airway organoids to study host factors involved in infection by coronaviruses. CRISPR-Cas9-driven gene editing was used to mutate host proteins exploited by the virus and identify putative therapeutic targets (Beumer et al. 2021). Such methodologies applied to CNS organoids and even organotypic cultures will surely be far more relevant than genetic screens and investigations performed in 2D cultures, because the former are more representative of the *in vivo* environment and pathogenesis, which should lead to findings that are clinically translatable.

In order to model viral neuroinfections even more pertinently, integrative *ex vivo* approaches should undoubtedly be implemented in the near future. Because neurological manifestations can also occur following peripheral infections, and virus-induced CNS damages can also impact peripheral organs, it is critical to develop models and tools to study inter-organ communication. A combination of organotypic cultures, organoids and organ-on-chips technologies may be among the future keys to explore such routes used by encephalitic viruses. The virology field widely adopted several techniques presented through this review, and pioneered some of them. The use of developing cutting-edge models is ongoing in the field, but maybe to a lower extent so far than in cancer research and developmental biology in particular, which are at the forefront for the implementation of these new systems (Karzbrun et al. 2018, Zhu et al. 2023). We believe that neurovirology research shall implement systems combining *ex vivo* models (e.g. organoid-on-chip, organotypic culture-on-chip, and even multiorgans-on-chip consisting of organoids and/or organotypic cultures from different organs on the same chip). This would allow to recreate a circulatory system and inter-organ communication in order to model neuroinfections in a quasi-physiological way. For example, to study the neuroinvasiveness of a neurotropic virus such as NiV or SARS-CoV-2, an organotypic lung culture could be combined with an OBC in a chip, with a BBB organoid at the interface, enabling the monitoring of the infection from the initial natural site of infection and the entry into the CNS. This would thus be advantageous to explore the remote events linked to these viruses, such as the influence of the permeabilization of the BBB, of its inflammation and that of peripheral organs, the remodeling of neurons at a distance, or the production of early markers implicated in the encephalitogenesis for instance. Adding organotypic liver cultures (Ogire et al. 2024) or liver organoids to the system could benefit antiviral and drug testing studies by mimicking the hepatic metabolism of drugs. Such models would complement the limits of each individual model, namely the fact that organoids are aggregates of self-assembled cells but are not originated from the organ itself, chips are an artificial construction, and organotypic cultures come from the organ itself but lack blood flow.

The latest developments of the OBC model for virology research consist notably in their use as a preclinical platform for antiviral studies combined with artificial intelligence. In a proof-of-concept study, electrical activity (local field potential more precisely, used as a proxy for neural health) was measured in human post-mortem OBCs placed on 3D microelectrode arrays upon infection with a neurotropic virus (Tahyna virus) and antiviral treatment. In this set-up, microelectrodes penetrate deep in the OBC, allowing relevant electrical measurement. Infection was shown to disturb local field potential, and machine learning was used to analyze antiviral efficiency via evaluation of OBC neurohealth based on electrical activity (Partiot et al. 2024a).

Keys to progress in neurovirology mainly reside in the transposition of tools from neurobiology. Among them, electromonitoring of OBCs is not a recent development. A dense literature reports the use of electrophysiological recordings in OBCs to characterize the model and study neuronal behaviors, brain processes, and diseases. Set-ups consisting of single tip electrodes inserted into the tissue, which can be used for electrical stimulation or recording, allow to investigate synaptic activity, neuron excitability, viability, and plasticity, while keeping the cells in their native environment (Stoppini et al. 1991, Dong and Buonomano 2005, Johnson and Buonomano 2009, Ting et al. 2018, Romero-Leguizamón et al. 2019, Bak et al. 2024). Three-dimensional microelectrode arrays, on which OBCs can be directly grown, have also been extensively optimized and enable larger-scale, multisite electrophysiological simultaneous recordings from a large number of neurons, including during long-term culture (Kristensen et al. 2001, Blake et al. 2010, Ito et al. 2014, Ravi et al. 2019, Romero-Leguizamón et al. 2019, Forro et al. 2021). Electromonitoring allows to address questions related to the impact of the infection on neuronal homeostasis, health, and activity, or whether a virus can leave a trace that can be detectable after the infection by analyzing the alterations of the electrical activity, for example.

These types of system are, for example, used in fundamental neuroscience to compare the neuronal network circuitry of different brain areas (Ito et al. 2014), in neuro-oncology to analyze neuronal activity and viability of human cortical OBCs (Ravi et al. 2019), or to investigate neuronal network activity and remodeling in the context of neurodegenerative diseases and other neuropathies (Croft et al. 2019, Bouillet et al. 2022). They are also applied to brain organoids, whose structure imposes the development of 3D electrophysiological devices covering or incorporated into the organoid to monitor the whole surface and the inner electrical activity (Passaro and Stice 2020, Forro et al. 2021, McDonald et al. 2023). For instance, a recently developed microelectrode array with protruding cantilevers that insert deeply into cerebral organoids allows access to internal neuronal cells (Phouphetlinthong et al. 2023). Other strategies consist of slicing cerebral organoids and culturing them at the air–liquid interface, similarly to OBCs, a technique that improves neuronal network viability and electrical activity (Giandomenico et al. 2019, Qian et al. 2020). However, the use of electromonitoring in CNS models is sorely lacking for the study of neuroinvasive and neurotropic viruses, even although it could be a key tool to decipher how infections impact the neural circuitry, neuronal and synaptic activity/plasticity, both in the early stages when the virus enters the CNS and in the long term.

Similarly, techniques that are commonly used in neuroscience research such as optogenetics shall be of interest for the study of CNS-targeting viruses in *ex vivo* models. The generation of photocontrollable neurotropic viruses, whose gene expression and replication can be temporally and spatially switched on and off by light, further supports this claim (Tahara et al. 2019, 2024). Robotic techniques could also be applied in the near future for precise micromanipulation of cells and microinjection of, for example, virus or specific molecules, for example, in specific cells, in the synaptic cleft or other specific areas of the CNS tissue, in 3D *ex vivo* models (Wong et al. 2014, Shull et al. 2019, 2021). In this way, these technologies could give significant insights into how viruses propagate between neural cells and impact their physiology, and that of specific compartments like synapses, for instance, as illustrated with SARS-CoV-2-induced perturbation of synaptic homeostasis (Partiot et al. 2024b), while also giving information on neural physiology itself.

Currently, the major limitations of the *ex vivo* models are that they mostly come from animals because of limited access to human samples, and that those of human origin generally entail an inherent variability. In addition, although protocols for preparation of human OBCs exist, the model is not as well standardized as animal model OBCs. Human OBCs can come from surgical resections, biopsies, post-mortem donors, or fetal samples, which means a certain degree of randomness in the areas of CNS being sliced and in the donor more globally. As an example, protocols to generate short-term human OBC maintained in liquid medium have been used for the study of Oropouche virus, an emerging neuroinvasive arbovirus that can induce neurological symptoms. In this model, mature human neural cells were shown to be susceptible to infection and to support the production of infectious virions. The main infected cells were microglia. Some neurons are also found infected, contrary to astrocytes. The release of pro-inflammatory tumor necrosis factor alpha was also noted upon infection, as well as an impairment of cell viability. A potential improvement could be to limit the hypoxia related to the immersion that can impact maintenance of microglia in the tissue and responses to the infection (Fernandes et al. 2019, Almeida et al. 2021). Regarding animal OBCs, reproducible and well-defined slices representative of the whole brain, from the olfactory bulb to the brain stem, or coronal slices, are still lacking, although attempts have been reported (Staal et al. 2011, Ullrich et al. 2011, Joost et al. 2017, McKenna et al. 2022, Uçar et al. 2022). Such a whole-brain model would be of great interest to investigate viral dissemination between cerebral substructures, susceptibility of the different substructures and cell types, global response of the cerebral tissue to the infection, virus evolution, for example, in a context where all the brain elements are present and still connected, and easily accessible for analysis.

## Concluding remarks

Through this review, we aimed at showing how CNS models can be used to study neuropathogenic viruses and to highlight key discoveries made thanks to them (Fig. 1 and Table 2). *Ex vivo* models are highly useful to better understand brain-targeting viruses, but these pathogens themselves (such as HSV or RABV) are also valuable tools with which to investigate CNS biology. The most obvious examples are their use for optogenetics, where they serve as vectors for the delivery of photoreceptors, or for neuronal tracing and, more generally, transgene expression. To date, organotypic cultures appear to be the most complex and physiologically relevant system, while being relatively easy to implement compared with organoids, for example. However, one must keep in mind that the *ex vivo* models of infection presented here are not representative *per se* of how infection naturally occurs *in vivo*. They give insights of what a virus is able to do (or not) in a specific CNS context, notably whether it can infect such models, when delivered in a non-physiological way. Putting the virus directly on neural, organotypic cultures, or organoids is not the same as an airborne virus, for example (such as MeV, NiV, or SARS-CoV-2), entering its host by its natural route of infection, and which possibly circulates through the body, infects target peripheral tissues, makes local and systemic damages, evolves, before potentially reaching the brain. This should thus be remembered when interpreting results and putting them in perspective with *in vivo* pathogenesis, to avoid over-speculation, and further supports the need for developing integrative multiorgans models. Considering the big picture, we perceive that while detailed molecular information regarding infection by neuroinvasive and neurotropic viruses is available in cell lines, non-*ex vivo* or non-neural models, the amount of data is to some extent sparser in more relevant CNS *ex vivo* models. Many studies focus their observations on the tissular and cellular scales, and less on the molecular one. Neurovirology research is somewhat late in integrating more advanced techniques and technologies to its models, compared with other fields of research, but has the limitation of dealing with infectious material. This is particularly true for BSL-3 and BSL-4 pathogens. There is still much to do to optimize and improve the *ex vivo* models presented throughout this review, notably in terms of standardization and characterization, but they warrant to be more widely used to obtain deeper molecular insight, beyond relatively basic questions, such as, for example, neurotropism. A major benefit of *ex vivo* models compared with *in vivo* experimentation that supports the incentive to see a greater use of these models is related to animal ethics, with drastic reduction of animal use, while preserving pertinent physiological contexts. Lastly, future combination of *ex vivo* models with artificial intelligence usage will surely allow better integration and correlation of the obtained data with the observations made *in vivo* and the pathogenesis descriptions in humans.

## Supplementary Material

fuaf024_Supplemental_Files
